# Synergistic Effects of Oxaliplatin, 5-Fluorouracil, and Novel Synthetic Uracil Analog U-359 on Breast Cancer Cell Carcinogenesis

**DOI:** 10.3390/ijms26072964

**Published:** 2025-03-25

**Authors:** Angelika Długosz-Pokorska, Tomasz Janecki, Anna Janecka, Katarzyna Gach-Janczak

**Affiliations:** 1 Department of Biomolecular Chemistry, Faculty of Medicine, Medical University of Lodz, Mazowiecka 6/8, 92-215 Lodz, Poland; 2 Institute of Organic Chemistry, Faculty of Chemistry, Lodz University of Technology, 92-215 Lodz, Poland

**Keywords:** MCF-7, oxalipaltin, 5-fluorouracil, U-359, anticancer compounds, apoptosis, multidrug resistance

## Abstract

Breast cancer presents significant global challenges, necessitating effective treatments to combat drug resistance and minimize chemotherapy side effects. This study evaluated the cytotoxic effects of U-359, Oxaliplatin (Ox), and 5-Fluorouracil (5-FU) in MCF-7 and MCF-10A cells using MTT and RealTime-GLO assays. Morphological changes were assessed by light microscopy following Wright–Giemsa staining. Apoptosis induction was studied using qPCR for apoptotic markers, the RealTime-Glo™ Annexin V assay, and the cleaved PARP1 ELISA assay. Caspase 8 and 9 activities, ABCB1, ABCG2, and NF-κB protein levels were quantified using ELISA. Synergy was analyzed using the Bliss Independence Model. The results indicated that combining U-359 with Ox and 5-FU enhanced cytotoxicity compared to individual treatments. U-359 induced apoptosis-associated morphological changes in MCF-7 cells, which were augmented with the Ox and 5-FU treatment. Apoptosis assays confirmed the up-regulation of pro-apoptotic markers and the down-regulation of anti-apoptotic markers with U-359 alone or in combination. Elevated cleaved PARP1 levels suggested robust apoptosis induction with U-359 and Ox or 5-FU. Caspase activity assays demonstrated a significant activation of caspase 8 and 9, implicating both apoptotic pathways. Furthermore, U-359 down-regulated ABCB1, ABCG2, and NF-κB in MCF-7 cells, which were up-regulated by Ox and 5-FU alone. The Bliss Independence Model revealed strong synergistic interactions (SI < 1) between U-359 and Ox or 5-FU, particularly in reducing ABCB1 and NF-κB levels. U-359 combined with Ox and 5-FU shows potential for overcoming chemotherapy resistance in breast cancer by enhancing apoptosis and modulating drug resistance. Further clinical studies are needed to optimize treatment and improve outcomes.

## 1. Introduction

Breast cancer remains a formidable global health challenge, characterized by rising incidence rates worldwide [1]. Despite significant advancements in treatment modalities such as surgery and pharmacotherapy, survival rates post-relapse remain suboptimal. This underscores the urgent need for innovative and more efficacious chemotherapeutic strategies. Currently, standard chemotherapies like doxorubicin, capecitabine, docetaxel, Taxol, gemcitabine, and vinorelbine yield moderate response rates in breast cancer, with limited progression-free survival periods [2,3,4,5,6].

Breast cancer cells frequently develop resistance mechanisms, significantly reducing drug efficacy and posing a major hurdle to successful chemotherapy. Overcoming drug resistance demands a comprehensive understanding of the complex molecular pathways contributing to multidrug resistance (MDR) phenotypes. Among others, nuclear factor κB (NF-κB) modulation and the overexpression of ATP-binding cassette (ABC) transporters are involved in this resistance [7,8]. Given the high toxicity and resistance associated with conventional cytotoxic agents, there is a critical need for novel therapeutic approaches.

Combination chemotherapy, involving the use of multiple drugs targeting distinct pathways, represents a promising strategy to enhance treatment efficacy while minimizing resistance development and side effects [9]. Therefore, novel compounds capable of synergizing with currently used drugs have constantly been sought and tested.

Uracil and its derivatives have attracted considerable attention due to their diverse biological activities. Initially identified as a component of ribonucleic acids in 1900, uracil has since served as the basis for numerous medicinal compounds [10]. The derivatives of uracil, such as 5-Fluorouracil (5-FU) and uramustine (uracil mustard), demonstrate anticancer properties and are used as chemotherapy drugs. 5-FU acts as a pyrimidine antimetabolite, inhibiting cell growth, while uramustine functions as both an antimetabolite and an alkylating agent. The simple structure of uracil allows the development of novel analogs with enhanced cytotoxic activity, such as 5-methylidenedihydrouracils, which incorporate a conjugated exo-methylidene double bond onto the dihydrouracil ring [11].

**Figure 1:**
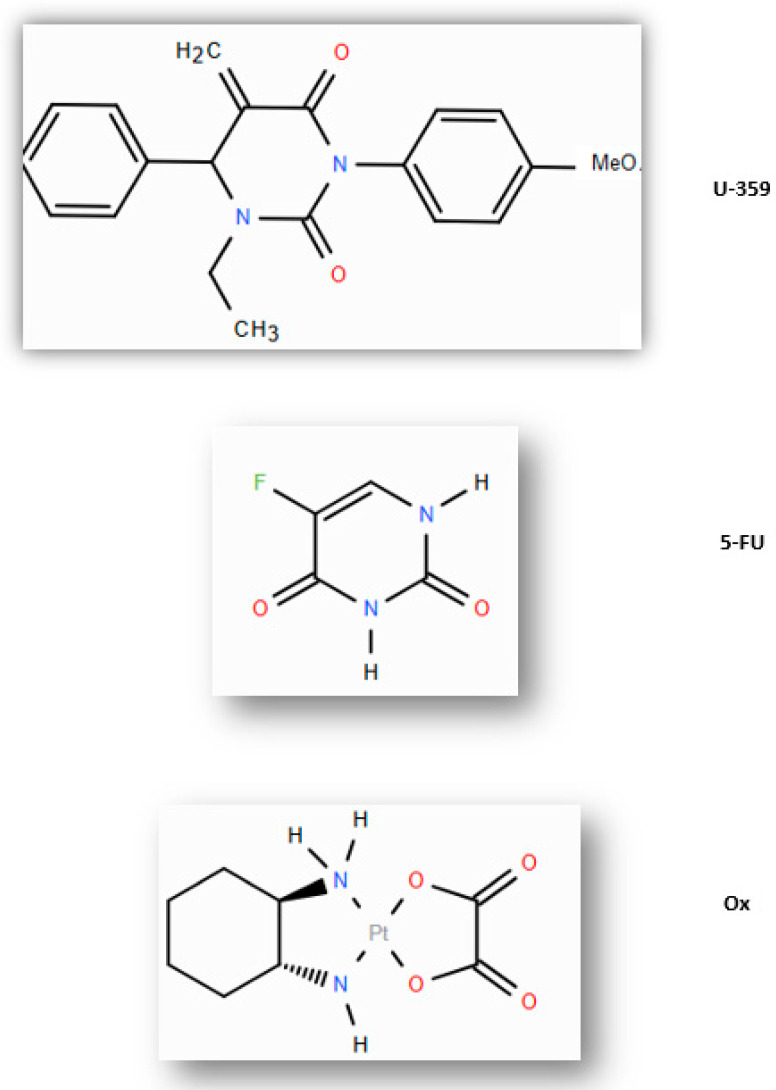
Chemical structures of U-359, 5-FU, and Ox.

In our previous study, we demonstrated the anticancer activity of a synthetic uracil analog, 3-p-bromophenyl-1-ethyl-5-methylidenedihydrouracil (U-359) (Figure 1) [12,13,14]. This compound inhibited MCF-7 cell proliferation and induced the apoptosis of cancer cells via the mitochondrial pathway. Furthermore, U-359 synergistically enhanced the anticancer effects of Taxol (Tx) and reversed Tx resistance in MCF-7 cells by stabilizing microtubules and modulating microtubule dynamics [12,13,14]. Our research also indicated that U-359 countered Tx-induced up-regulation of ABCB1, ABCG2, and NF-κB proteins, suggesting its potential in overcoming MDR. This dual modulatory role makes U-359 a promising candidate for combination therapy enhancing the efficacy of breast cancer treatments [15].

Why was continuing this research essential?:

1. Unanswered questions from previous research.
  While our initial studies provided promising evidence that U-359 could enhance the effects of Tx, they also raised critical questions:
  - Does U-359 act specifically against microtubule-targeting drugs like Taxol, or does it have broader anticancer potential?
  - Would U-359 have similar synergistic effects with other widely used different chemotherapeutic agents?
2. The need for more effective strategies to overcome MDR in cancer cells.
  - Ox resistance often develops due to DNA repair mechanisms and anti-apoptotic signaling.
  - 5-FU resistance is frequently linked to the up-regulation of NF-κB and ABC transporters.
  - Both drugs suffer from declining efficacy over time, limiting their long-term clinical success.
3. Changing and expanding the possible therapeutic potential of U-359.
  If U-359 only worked with Taxol, its use would be limited to certain cancer types. However, if it could also enhance Ox and 5-FU therapy, it could be used in a much wider range of treatments, including the following:
  - Breast cancer (where Ox and 5-FU are standard treatments).
  - Colorectal cancer (5-FU is a first-line therapy).
  - Ovarian cancer (Ox is widely used).
4. Optimizing our strategies by reducing toxicity and increasing efficacy.
  Traditional chemotherapy is associated with severe toxicity due to the high doses required for effectiveness. If U-359 could enhance the efficacy of Ox and 5-FU, it could allow for the following:
  - Lower chemotherapy doses, reducing side effects such as nausea, immune suppression, and organ toxicity.
  - More effective cancer cell killing at lower drug concentrations, improving patient outcomes.
  - A safer treatment alternative for patients who cannot tolerate high-dose chemotherapy.
5. The need for mechanistic validation.
  While our previous studies showed that U-359 had anticancer effects, we needed to validate the precise molecular mechanisms by which it enhances chemotherapy.
  Without this additional research, we would not have fully understood how U-359 works, limiting its clinical potential.
  Continuing this research was not just beneficial—it was essential to do the following:
  - Determine whether U-359’s effects were limited to Taxol or had broader applications.
  - Validate its ability to overcome drug resistance in Ox- and 5-FU-treated cancer cells.
  - Confirm that U-359 allows for chemotherapy dose reduction, improving safety.
  - Provide mechanistic insights into how U-359 enhances apoptosis and suppresses MDR. For this purpose, we studied the influence of U-359, used alone or in combination with 5-FU or Ox, on apoptosis induction and the inhibition of MCF-7 cell proliferation. We also analyzed the potential of U-359 as an ABC transporter inhibitor and NF-κB modulator.

This study was designed to understand the mechanism by which U-359 can augment standard chemotherapy and reduce drug resistance, paving the way for innovative breast cancer treatments. Without these additional studies, the full potential of U-359 as a universal chemosensitizer would have remained unknown. Our findings pave the way for future clinical research, bringing us one step closer to a more effective and personalized approach to breast cancer treatment.

## 2. Results

### 2.1. Cytotoxic Activity

**Figure 2:**
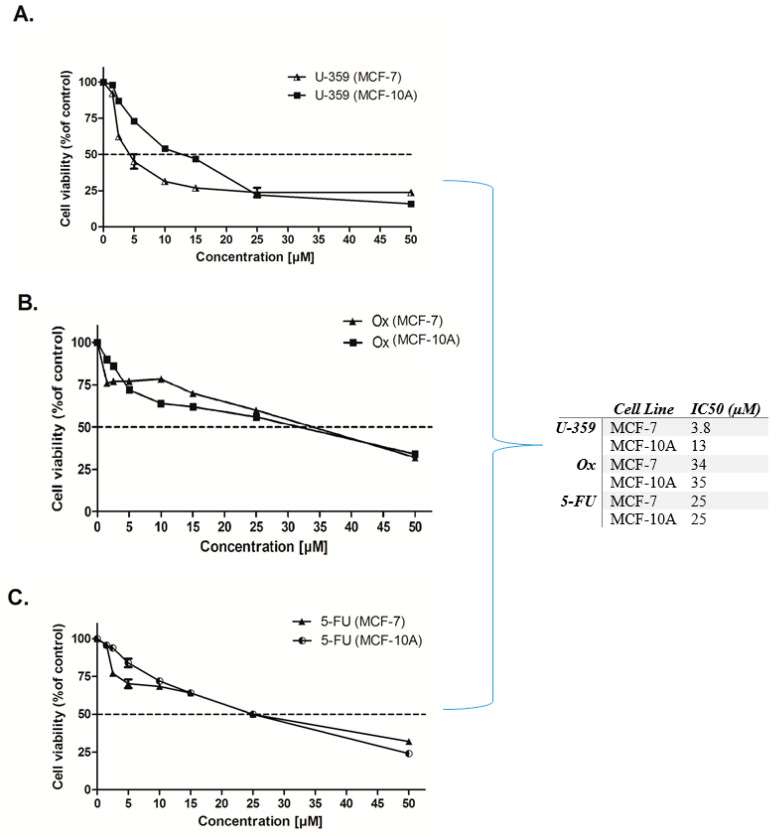
The cytotoxic effect of U-359 (**A**), Ox (**B**), and 5-FU (**C**) in MCF-7 and MCF-7-10A cells, analyzed by MTT assay. Each data point represents the mean of three replicates, and the error bars indicate SEM.

The cytotoxic effects of the tested compounds were evaluated in both cancerous MCF-7 and non-tumorogenic MCF-10A cells, using the standard MTT assay. This assay measures cell viability based on the ability of living cells to convert the yellow, water-soluble tetrazolium salt into an insoluble purple formazan product. U-359 exhibited significant cytotoxicity in MCF-7 cells, with an IC_50_ value of 3.8 μM. This compound was about three-fold more toxic for MCF-7 cells, as compared with MCF-10A cells, where IC_50_ was 13 μM [14]. The cytotoxic activity of Ox and 5-Fu was almost identical for cancer and non-cancerogenous cells. In MCF-7 cells, the IC_50_ values were 34 and 25 μM, whereas in MCF10A, 35 and 25 μM, for Ox and 5-FU, respectively (Figure 2). The concentration ranges and IC_50_ values for 5-FU and Ox were comparable with those reported in the literature [16,17].

### 2.2. Analysis of Cell Morphology Using Light Microscopy

**Figure 3:**
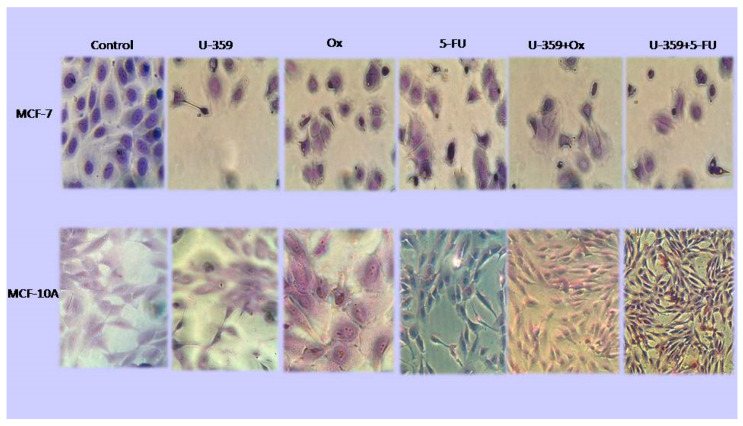
Effect of U-359, Ox, 5-FU, U-359+Ox, and U-359+5-FU on the morphology of MCF-7 and MCF-10A cells after 24 h of incubation. After staining with Giemsa dye, the cells were photographed under a light microscope with a built-in camera (magnification 100×).

Morphological changes in MCF-7 and MCF-10A cells treated with U-359, Ox, 5-Fu, or combination (U-359+Ox and U-359+5-FU) for 24 h were determined using Wright–Giemsa staining (Figure 3). The control, untreated MCF-7, and MCF-10A cells displayed a normal angular or polygonal shape, with a large vesicular nucleus and prominent nucleolus. In MCF-7 cells, treatment with U-359, Ox, or 5-FU led to noticeable changes, including cell enlargement, chromatin condensation, nuclear fragmentation, and the formation of small apoptotic bodies. In combined treatments (Ox+U-359 or 5-FU+U-359), a significant increase in the number of cells exhibiting apoptotic features was observed. In contrast, MCF-10A cells treated with U-359 retained the normal morphology of MCF-10A cells. Incubation with Ox or 5-FU caused significant abnormal morphological changes. Cells treated with U-359+Ox or U-359+5-FU displayed a mix of normal and abnormal morphology.

### 2.3. Effect of Single and Combined Treatments on MCF-7 and MCF-10A Cell Viability

To evaluate the impact of a single (U-359, Ox, and 5-Fu) or combined treatments (U-359+Ox and U-359+5-FU) on cell viability in MCF-7 and MCF-10A cell lines after 24 h of incubation, the RealTime-GLO MT Cell Viability Assay (Promega, Walldorf, Germany) was utilized. In this assay, healthy cells metabolize the proprietary pro-substrate to produce a substrate for NanoLuc^®^ luciferase (Promega, Walldorf, Germany), which diffuses into the culture medium, resulting in a luminescent signal proportional to the number of viable cells. The efficacy of combination treatments was compared to individual treatments.

**Figure 4:**
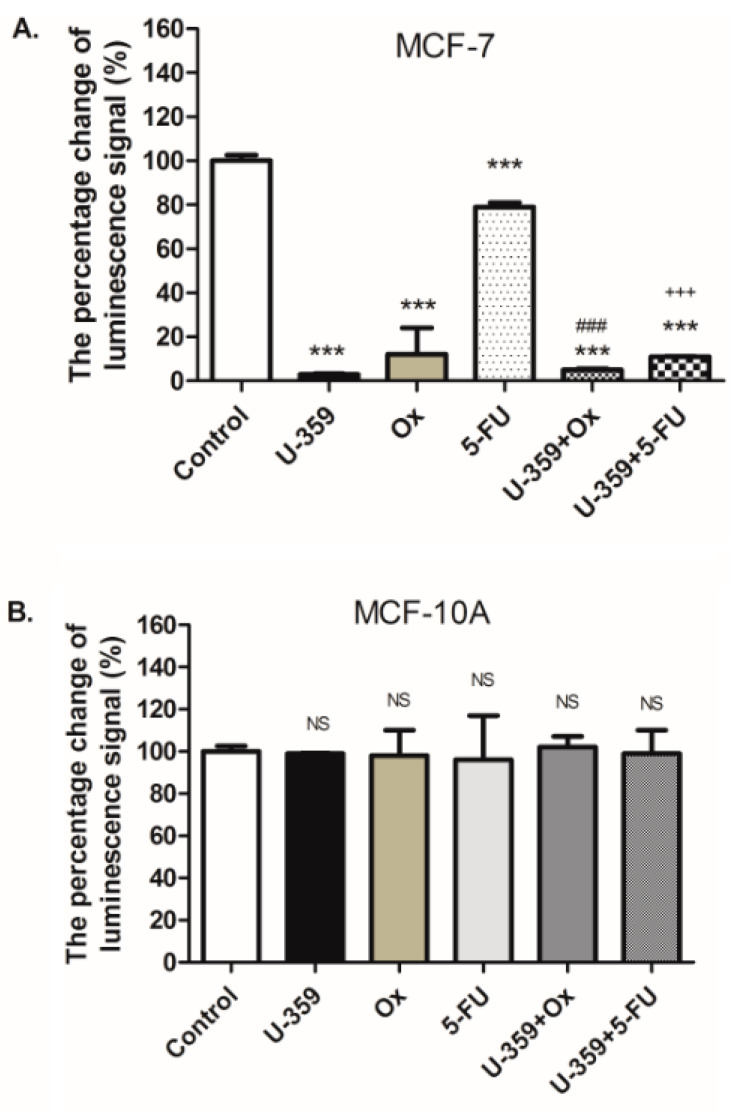
Effect of U-359 (3.8 μM for MCF-7 and 13 μM for MCF-10A), Ox (34 μM for MCF-7 and 35 μM for MCF-10A), 5-FU (25 μM for both cell lines), U-359+Ox, and U-359+5-FU on MCF-7 and MCF-10A cell viability, expressed as the percentage change in luminescence signal (%), after 24 h of incubation (**A**,**B**). Data are expressed as mean ± SEM. Statistical significance was assessed using one-way ANOVA and a post hoc multiple comparison Student–Newman–Keuls test. *** *p* < 0.001, in comparison with control; ^###^ *p* < 0.001, in comparison with Ox; ^+++^ *p* < 0.001, in comparison with 5-FU; NS, not significant.

In MCF-7 cells, the compound U-359 demonstrated the most potent effect, significantly reducing cell viability by 97%. When treated with Ox or 5-FU, cell viability was reduced by 88% and 21%, respectively (as shown in Figure 4). Notably, combined treatments with U-359+Ox or U-359+5-FU further diminished cell viability, reaching only 95% and 89% of the viability levels observed with Ox and 5-FU as single agents, respectively. This indicates a synergistic effect of the combined treatments, leading to a more pronounced decrease in MCF-7 cell viability compared to individual drug treatments. In MCF-10A cells, neither individual nor combined treatments produced significant changes in cell viability.

Using the synergy index (SI) method [18], the synergy of the treatment combinations was evaluated. The obtained results revealed a synergistic effect for both Ox+U-359 (SI = 0.562) and 5-FU+U-359 (SI = 0.871) combinations, indicating the potential of U-359 to enhance the efficacy of Ox and 5-FU.

### 2.4. Apoptosis Analysis

#### 2.4.1. Analysis of Apoptosis-Related Genes Expression

The mRNA levels of the *Bax*, *Bcl-2*, *p21*, *p53*, and *Caspase 3* genes were assessed in MCF-7 cells after 24 h of exposure to single (U-359, Ox, and 5-Fu) or combined treatments (U-359+Ox and U-359+5-FU) using a real-time PCR assay. Treatment with U-359 alone significantly increased the expression of *Bax*, *p21*, *p53*, and Caspase 3 genes and decreased *Bcl-2* gene expression levels in comparison to the control. On the other hand, incubation with Ox or 5-FU alone led to the down-regulation of Bax, p21, and p53 gene expression. Interestingly, Ox treatment up-regulated *Bcl-2* and *Caspase 3* expression, while 5-FU treatment resulted in *Bcl-2* up-regulation and *Caspase 3* down-regulation.

The combined treatment U-359+Ox, as well as U-359+5-FU, significantly increased *Bax*, *p53*, *Caspase 3*, and *p21* gene expression and decreased *Bcl*-2, compared to the Ox or 5-FU treatment alone (Table 1).

#### 2.4.2. Assessment of Apoptosis and Necrosis

The RealTime-Glo™ Assay was employed to distinguish between apoptotic and necrotic cell death induced in MCF-7 cells after 24 h of single (U-359, Ox, and 5-Fu) or combined (U-359+Ox and U-359+5-FU) treatments. Apoptosis was detected through an increase in the luminescence signal level, which indicates the externalization of phosphatidylserine to the outer surface of the cell membrane. Necrosis was identified by an elevated fluorescence signal level, which signals the presence of phosphatidylserine on both the inner and outer cell membranes. An increase in both luminescence and fluorescence signal levels suggested a loss of membrane integrity, indicative of secondary necrosis following apoptosis. Conversely, a high luminescence signal level without an increase in fluorescence indicated early apoptosis, associated with Annexin V binding to phosphatidylserine on the outer cell membrane.

**Figure 5:**
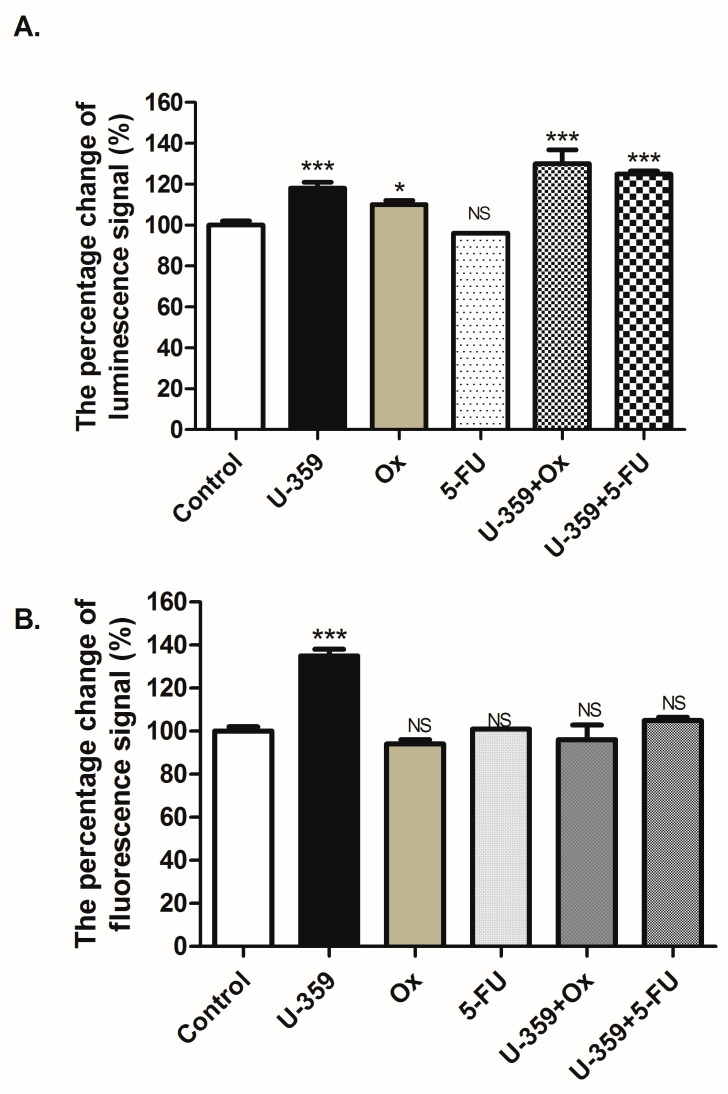
Effect of U-359, Ox, 5-FU, U-359+Ox, and U-359+5-FU on induction of apoptosis and/or necrosis in MCF-7 after 24 h of treatment, measured by the changes in luminescence (**A**) and fluorescence (**B**) signals. Data are expressed as mean ± SEM. Statistical significance was assessed using one-way ANOVA and a post hoc multiple comparison Student–Newman–Keuls test. *** *p* < 0.001, * *p* < 0.05 in comparison with control; NS, not significance.

As shown in Figure 5, the incubation of MCF-7 cells with U-359 resulted in a 35% increase in fluorescence and an 18% increase in luminescence, in comparison to the control, which indicates secondary necrosis following apoptosis. The treatment of MCF-7 cells with 5-FU alone did not significantly affect fluorescence or luminescence, while Ox caused a slight 7% increase in luminescence compared to the control. When MCF-7 cells were treated with U-359+Ox or U-359+5-FU, the fluorescence signal remained unchanged, while luminescence increased significantly by 30% for U-359+Ox and 26% for U-359+5-FU, indicating the induction of early apoptosis. The synergistic effect was confirmed by calculated synergy indices, SI = −0.29 for U-359+Ox and SI = 0.24 for U-359+5-FU.

#### 2.4.3. Evaluation of Human PARP1 Protein Levels Using an ELISA-Based Approach

The effects of single (U-359, Ox, and 5-Fu) or combined (U-359+Ox and U-359+5-FU) treatments on apoptosis induction in MCF-7, after 24 h of incubation, were assessed using the Cleaved PARP1 Human SimpleStep ELISA^®^ Kit (ABCAM, Cambridge, UK). Poly ADP-ribose polymerase 1 (PARP1) is a DNA repair enzyme that is cleaved by activated caspases during apoptosis. The cleaved form, 89 kDa PARP1, is thus considered a hallmark of apoptosis.

**Figure 6:**
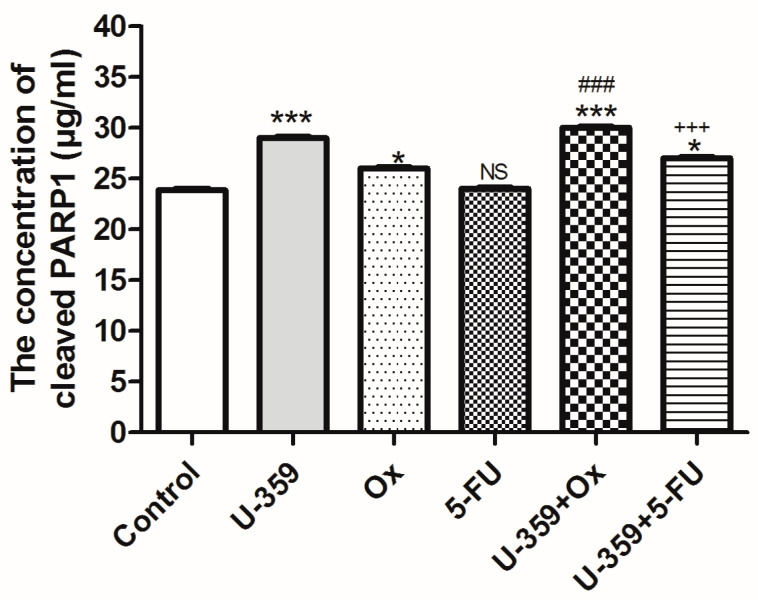
Effect of U-359, Ox, 5-FU, U-359+Ox, and U-359+5-FU on concentration of cleaved PARP1 in MCF-7 cells after 24 h of treatment, measured by ELISA-based method using Cleaved PARP1 Human SimpleStep ELISA^®^ Kit. Data are expressed as mean ± SEM. Statistical significance was assessed using one-way ANOVA and a post hoc multiple comparison Student–Newman–Keuls test. * *p* < 0.05 and *** *p* < 0.001, in comparison with control; ^###^ *p* < 0.001, in comparison with Ox; ^+++^ *p* < 0.001, in comparison with 5-FU. NS, not significant.

As shown in Figure 6, the treatment of MCF-7 cells with U-359 or Ox increased the levels of cleaved 89 kDa PARP1 by 22% and 7%, respectively, whereas 5-FU did not produce a significant effect. However, in combined experiments where MCF-7 cells were treated with U-359+Ox or U-359+5-FU, the levels of cleaved 89 kDa PARP1 increased by 24% and 12%, respectively, compared to the effects produced by Ox and FU-5 alone.

The synergy indices indicated that the combination of U-359 with Ox (SI = 0.84) and with 5-FU (SI = 0.52) both displayed synergistic interactions, with the former exhibiting a more pronounced effect.

#### 2.4.4. The Caspase 8 and 9 Activity

To measure the activity of Caspase 8 and Caspase 9 in MCF-7 cells after 24 h of single (U-359, Ox, and 5-Fu) or combined (U-359+Ox and U-359+5-FU) treatments, the Caspase-Glo 8 and 9 luminometric assay kit was utilized. Caspase 8 is crucial in the extrinsic apoptotic pathway, while Caspase 9 is essential for mitochondrial remodeling during intrinsic apoptosis. In the Caspase-Glo 8 and 9 assay, the luminescence signal, produced upon the cleavage of luminescent Caspase 8 and 9 reagents, is directly proportional to the level of caspase activity.

**Figure 7:**
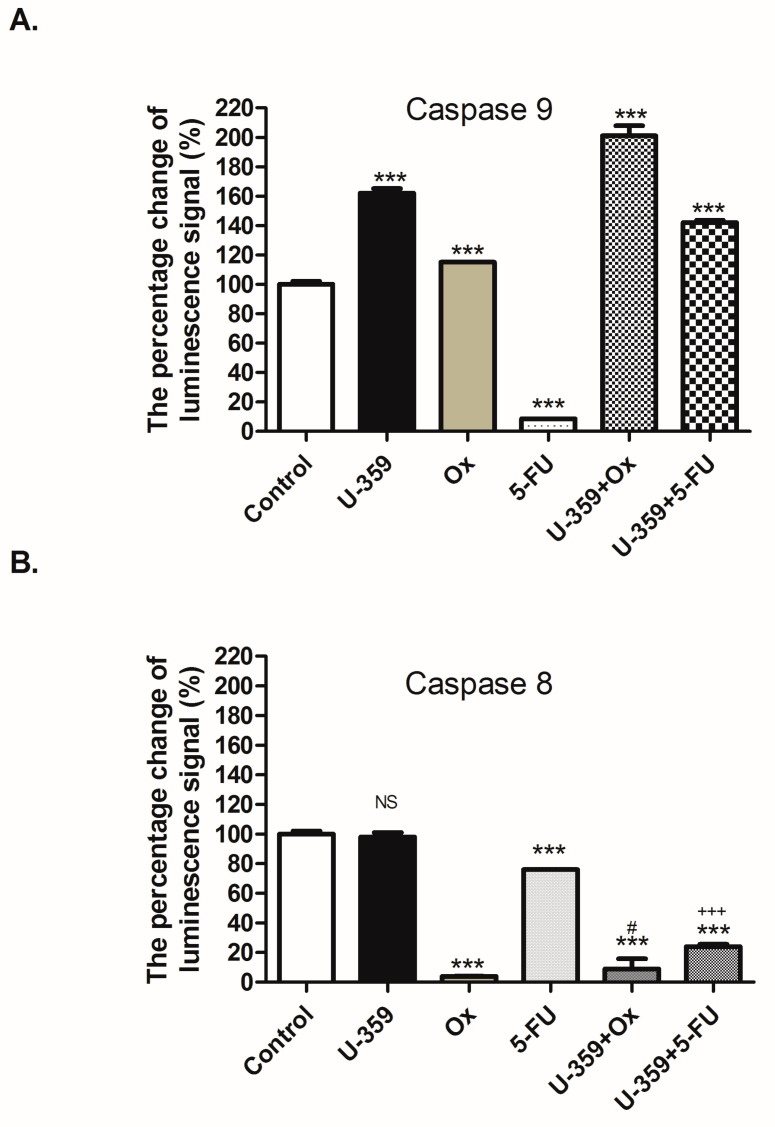
Effect of U-359, Ox, 5-FU, U-359+Ox, and U-359+5-FU on Caspase 9 (**A**) and 8 (**B**) activity in MCF-7 cells after 24 h of treatment, measured by the percentage change in luminescence signal (%). Data are expressed as mean ± SEM. Statistical significance was assessed using one-way ANOVA and a post hoc multiple comparison Student–Newman–Keuls test. *** *p* < 0.001, in comparison with control; ^#^ *p* < 0.05, in comparison with Ox; ^+++^ *p* < 0.001, in comparison with 5-FU. NS, not significant.

As demonstrated in Figure 7, U-359 significantly increased the Caspase 9 activity by approximately 62% compared to the control group, without activating Caspase 8. In contrast, treatment with 5-FU decreased the activity of both Caspase 8 (by 75%) and Caspase 9 (by 8%). The incubation of MCF-7 cells with Ox resulted in a 15% increase in Caspase 9 and a 96% decrease in Caspase 8 activity. Importantly, the combined treatment of MCF-7 cells with U-359+Ox or U-359+5-FU led to a substantial increase in Caspase 9 activity by more than 100% compared to treatment with Ox or 5-FU alone. The synergy indices confirmed the presence of a synergistic effect. The combinations of U-359 either with Ox or 5-FU demonstrated synergy in relation to Caspase 9, with SI values of 0.01 and 0.67, respectively. Notably, both U-359+Ox and U-359+5-FU exhibited negative synergy with Caspase 8, suggesting a potential antagonistic interaction.

### 2.5. Multidrug Resistance Phenotype

#### 2.5.1. Analysis of Multidrug Resistance-Related Genes Expression

The mRNA expression levels of the ABCB1, ABCG2, and NF-κB genes were examined in MCF-7 cells after 24 h of single (U-359, Ox, and 5-FU) or combined (U-359+Ox and U-359+5-FU) treatments, using real-time PCR. Upon treatment with U-359, the expression of ABCB1, ABCG2, and NF-κB displayed a remarkable decrease compared to untreated cells, which is in accordance with our previous study. On the other hand, the incubation of MCF-7 cells with Ox or 5-FU resulted in the up-regulation of all the tested gene expressions compared to the control, with the most pronounced effect for Ox. The up-regulation of multidrug resistance-related genes was reversed when Ox and 5-FU were used in combination with U-359 (Table 2).

#### 2.5.2. Regulation of ABCB1, ABCG2, and NF-κB p65 Proteins

**Figure 8:**
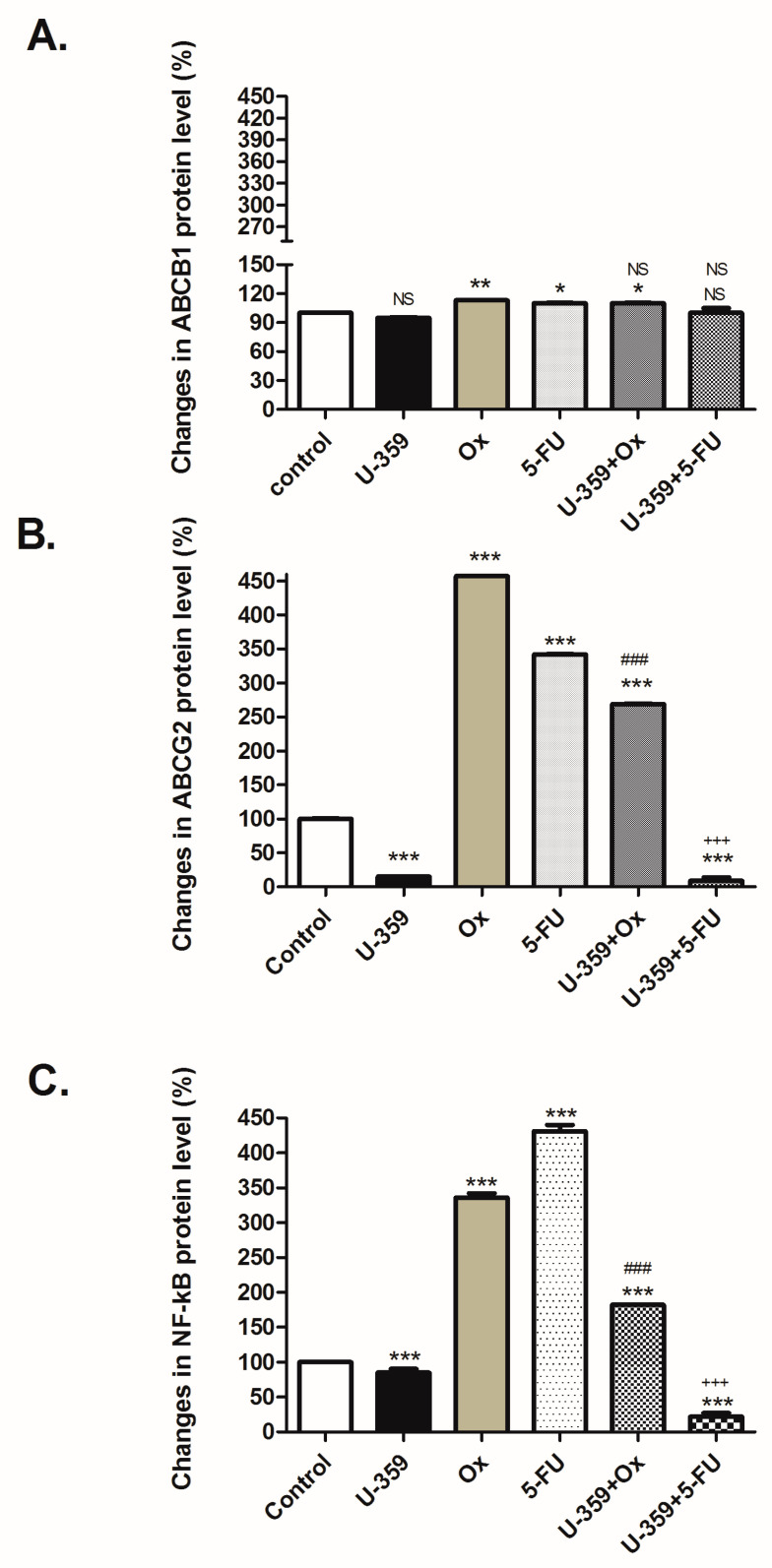
Effect of U-359, Ox, 5-FU, Ox+U-359, and 5-FU+U-359 on ABCB1 (**A**) and ABCG2 (**B**) and NF-κB (**C**) protein levels in MCF-7 cells after 24 h of incubation, measured by ELISA-based method. Data are presented as mean ± SEM. In each experiment, three replicates were used. The control consisted of untreated MCF-7 cells. Statistical significance was assessed by one-way ANOVA and a post hoc multiple comparison Student–Newman–Keuls test. *** *p* < 0.001, ** *p* < 0.01 and * *p* < 0.05, in comparison with control; ^###^ *p* < 0.001, in comparison with Ox; ^+++^ *p* < 0.001, in comparison with 5-FU. NS, not significant changes.

The concentrations of ABCB1, ABCG2, and NF-κB p65 proteins in MCF-7 cells after 24 h of treatment with U-359, Ox, 5-FU, and their combinations (Ox+U-359; 5-FU+U-359) were evaluated using ELISA kits, according to the protocol detailed in the Methods section. MCF-7 cells treated with U-359 displayed a significant decrease in ABCG2 and NF-κB protein levels in comparison to the control. Aligning with previously published data, U-359 did not significantly influence ABCB1 transporter expression (Figure 8).

The treatment of MCF-7 cells with Ox or 5-FU resulted in a slight elevation of ABCB1 protein and strong up-regulation of ABCG2 and NF-κB protein levels, which may be associated with the development of drug resistance mechanisms. These up-regulated ABCG2 and NF-κB protein levels were decreased by the combined treatment of U-359+Ox or U-359+5-FU; however, such treatment did not influence the level of ABCB1 protein. The synergy indices confirmed the presence of a synergistic effect (Table 3).

## 3. Discussion

Breast cancer is the second leading cause of cancer death in women. Traditional chemotherapy primarily relying on cytotoxic drugs is associated with high toxicity and the risk of drug resistance development. The search for novel innovative therapeutic strategies to overcome these problems is a challenge in breast cancer treatment. A promising approach gaining attraction is combination chemotherapy, which utilizes at least two drugs with distinct mechanisms of action. This strategy significantly lowers the chances of cancer cells developing resistance. Additionally, employing drugs that target multiple signaling pathways enhances the overall efficacy of treatment, enabling the administration of each drug at its optimal dose and thereby minimizing adverse effects. The urgent need for novel, highly specific antitumor treatments drives researchers to explore new compounds that, when combined with existing antineoplastic agents, could significantly improve chemotherapy outcomes [18].

Following this direction, in our previous articles, we described anticancer activity and synergistic effects with Tx of a novel synthetic uracil analog, 3-p-bromophenyl-1-ethyl-5-methylidenedihydrouracil (U-359) [12,13,14]. Continuing our research on U-359, in this study, we investigated the synergistic potential of this compound in combination with two established chemotherapeutic agents, Ox and 5-FU in MCF-7 cells. These chemotherapeutic drugs are widely used for the treatment of various solid tumors, including breast cancer. Ox is a third-generation platinum-based chemotherapeutic agent, similar to cisplatin and carboplatin. Ox exerts its cytotoxic effects by forming both inter- and intra-strand DNA crosslinks, which inhibit DNA replication and transcription, leading to cell cycle arrest and apoptosis [19]. 5-FU is a nucleoside analog, which inhibits thymidylate synthase, thereby disrupting DNA synthesis and leading to the accumulation of DNA damage, including double-stranded DNA breaks (DSBs) [20]. Additionally, 5-FU can induce apoptosis through mechanisms involving the modulation of the Bax/Bcl-2 or Bcl-xl ratio, further enhancing its cytotoxic effects against cancer cells. Despite the effectiveness, the clinical utility of Ox and 5-FU is limited by their potential for severe side effects and the development of resistance over prolonged use [21].

Our findings strongly indicate that U-359 potentiates the apoptotic effects of both Oxaliplatin (Ox) and 5-Fluorouracil (5-FU) in breast cancer cells. This was evidenced by a significant increase in Caspase 3/9 activity and elevated cleaved PARP1 levels, both of which are hallmark indicators of apoptotic cell death. Caspases, particularly Caspase 3 and 9, are central mediators of the intrinsic apoptotic pathway, and their activation leads to the breakdown of key cellular components, such as PARP1, which is crucial for DNA repair. The elevation of cleaved PARP1 suggests that U-359 enhances the intrinsic apoptosis cascade, sensitizing cancer cells to chemotherapy-induced cell death. These findings are consistent with previous studies that demonstrate the role of apoptotic modulation in overcoming chemoresistance and improving therapeutic outcomes.

An important aspect of our study is the dual modulatory effect of U-359 on the drug resistance mechanisms that often limit the efficacy of Ox and 5-FU. Specifically, we observed that U-359 down-regulates the expression of key multidrug resistance (MDR) proteins, including ABCB1 (P-glycoprotein), ABCG2 (breast cancer resistance protein), and NF-κB (nuclear factor kappa-light-chain-enhancer of activated B cells). These proteins are critical contributors to the efflux of chemotherapeutic agents from cancer cells, thereby reducing their intracellular concentrations and promoting resistance. ABCB1 and ABCG2, in particular, are known to confer resistance to a wide range of chemotherapy drugs by actively pumping them out of cells. NF-κB, a transcription factor, is involved in regulating the expression of genes related to cell survival, inflammation, and drug resistance. By down-regulating these key resistance markers, U-359 appears to mitigate the cellular defense mechanisms that typically prevent Ox and 5-FU from achieving their full therapeutic potential.

The modulation of these resistance pathways is particularly noteworthy, as it suggests that U-359 may reverse or circumvent the intrinsic chemoresistance mechanisms in breast cancer cells. This modulation could sensitize tumor cells to conventional chemotherapy, potentially improving the overall therapeutic response. Moreover, the synergistic effects of U-359 with Ox or 5-FU, observed in both in vitro and ex vivo models, provide further evidence of the potential benefits of this combinatorial approach. The synergy between U-359 and these chemotherapeutic agents was quantitatively validated using the Bliss Independence Surface Model, which demonstrated robust synergism, especially in the reduction in ABCG2 and NF-κB protein levels. This synergy suggests that U-359 not only enhances the apoptotic response to chemotherapy but also optimizes the pharmacological efficacy of these agents. The ability to achieve synergistic interactions between U-359 and chemotherapy agents is critical, as it enables the use of lower, more tolerable doses of chemotherapy while still maintaining or even enhancing its therapeutic effects. This strategy could minimize the adverse side effects commonly associated with high-dose chemotherapy, improving the quality of life for patients undergoing treatment.

In summary, our study presents compelling evidence supporting the potential of U-359 as a potent modulator of breast cancer therapy, particularly in combination with Ox and 5-FU. By enhancing apoptotic pathways and modulating key drug resistance mechanisms, U-359 offers a promising approach to overcoming chemotherapy resistance, a major barrier to effective cancer treatment. The combination of U-359 with standard chemotherapy could provide a valuable adjunct to current therapeutic regimens, offering new hope for patients with breast cancer who are resistant to conventional treatments.

Furthermore, the mechanistic insights gained from this investigation, particularly regarding the modulation of apoptotic signaling and MDR markers, provide a deeper understanding of how U-359 exerts its effects at the molecular level. These findings lay the groundwork for future studies aimed at evaluating the efficacy of U-359 in breast cancer cell lines resistant to Ox and 5-FU, such as MCF-7 cells with acquired resistance. Moreover, the promising results of this study should encourage the exploration of U-359’s applicability across different cancer types. Given the role of ABCB1, ABCG2, and NF-κB in multidrug resistance across various malignancies, U-359 may hold potential as a broad-spectrum adjunct to chemotherapy, warranting further investigation in preclinical and clinical settings.

Future studies will also aim to explore the pharmacokinetics and pharmacodynamics of U-359 in vivo, assess its toxicity profile, and investigate its potential synergistic effects in combination with other novel anticancer agents. By addressing these aspects, we can better understand how to optimize U-359-based therapies, paving the way for their eventual clinical application and improving treatment outcomes for patients with chemotherapy-resistant cancers.

## 4. Materials and Methods

### 4.1. Materials

The synthesis of U-359 followed established protocols [12]. Ox and 5-FU were purchased from Merck (Germany). For experimental preparations, U-359, Ox, and 5-FU were initially dissolved in DMSO and subsequently diluted in a culture medium, ensuring a DMSO concentration of less than 0.1%.

### 4.2. Cell Culture

MCF-7, a human breast adenocarcinoma cell line, was procured from the European Collection of Cell Cultures (ECACCs). The cells were cultured in Minimum Essential Medium Eagle (MEME), supplemented with non-essential amino acids, 2 mM glutamine, and antibiotics (100 mg/mL streptomycin and 100 U/mL penicillin), all sourced from Merck (Darmstadt, Germany). Additionally, the culture medium was supplemented with 10% fetal bovine serum from Biological Industries (Beit-HaEmek, Israel). MCF-10A, a non-tumorigenic human mammary breast cell line, was acquired from the American Type Culture Collection (ATCC). These cells were cultured using the MEGM Mammary Epithelial Bullet Kit purchased from Lonza (Walkersville, MD, USA). Both cell lines were maintained at 37 °C in a 5% CO_2_ atmosphere and were cultured until reaching 80% confluence.

### 4.3. MTT-Assay

The MTT assay, employing 3-(4,5-dimethylthiazol-2-yl)-2,5 diphenyl tetrazolium bromide as the substrate, was conducted to evaluate cellular dehydrogenase activity [22].

### 4.4. Determination of Cell Morphology Using Light Microscopy

For the detection of apoptosis and necrosis in MCF-7 and MCF-10A cells, Wright and Giemsa staining (Merck KGaA, Darmstadt, Germany) was employed. Giemsa dye imparts an orange-to-pink hue to the cytoplasm and a blue-to-purple shade to the nucleus, contingent upon the cytoplasmic contents’ acidity.

In brief, cells were seeded in 6-well plates (4.0 × 10^5^ cells/well) and cultured for 24 h with U-359, Ox, 5-Fu alone, or with U-359 in combination with Ox or 5-FU at their respective IC_50_ concentrations. Untreated cells served as negative controls. Following the incubation period, the medium was aspirated, and cells were fixed in methanol for 5 min, rinsed with PBS, and stained with the Wright and Giemsa solution for 30 min. Afterward, cells were washed twice with H_2_O and allowed to air dry. Finally, the stained cells were observed under a light microscope and photographed.

### 4.5. RealTime-Glo MT Cell Viability Assay

For assessing the cell viability of MCF-7 and MCF-10A cells, the RealTime-Glo™ MT (RTG MT) assay (Promega, Mannheim, Germany) was carried out following the standard protocol. Initially, MCF-7 and MCF-10A cells were seeded in 96-well plates at a density of 0.5 × 10^4^ cells/mL in 50 μL of standard growth medium and incubated for 24 h with U-359, Ox, 5-Fu alone, or with U-359 in combination with Ox or 5-FU at their respective IC_50_ concentrations. Subsequently, 50 μL of 2× RealTime-Glo reagent was added to each well after the incubation period. The plate was then incubated for 1 h at 37 °C in a humidified incubator. The luminescence signal intensity was measured using Flexstation 3. The efficacy of the combination treatment was compared to that of the individual compounds tested alone.

### 4.6. Assessment of Cell Death Type

The cell death type was determined using the RealTime-GloTM Annexin V Apoptosis and Necrosis Assay (Promega, Mannheim, Germany), following the manufacturer’s instructions. The assay kit comprised Annexin V-LgBiT (1000×) (Promega, Mannheim, Germany), Annexin V-SmBiT (1000×) (Promega, Mannheim, Germany), CaCl_2_ (1000×) (Promega, Mannheim, Germany), Annexin V NanoBiT^®^ Substrate (1000×) (Promega, Mannheim, Germany), and Necrosis Detection Reagent (1000×) (Promega, Mannheim, Germany).

In brief, MCF-7 cells were seeded in 96-well plates at a density of 1.0 × 10^4^/mL in 50 μL of standard growth medium and incubated for 24 h with U-359, Ox, 5-Fu alone, or with U-359 in combination with Ox or 5-FU, at their respective IC_50_ concentrations. Following the incubation period, 100 μL of 2× detection reagent was added to each well. The plate was then shaken for 30 s (at 500–700 rpm) and placed in a humidified incubator. Luminescence signal and fluorescence intensity were measured using Flexstation 3. The impact of combination treatments was compared to that of individual compound treatments.

### 4.7. Assessment of Human PARP1 Protein Levels by ELISA-Based Method

To determine the PARP1 protein level in MCF-7 cells exposed to the tested compounds, the Cleaved PARP1 Human Simple Step ELISA^®^ Kit (ABCAM, Cambridge, UK) was utilized. Cells were plated in 6-well plates and treated with U-359, Ox, 5-Fu alone, and with U-359 in combination with Ox or 5-FU at their IC_50_ concentrations for 24 h. Following treatment, the growth medium was aspirated, and cold 1× Cell Extraction Buffer PTR was added directly to the plates. After a 15 min incubation on ice, cellular lysates were centrifuged, and 25 μg of properly diluted protein extracts were added to antibody-coated 96-well plates. Cleaved PARP1 bound to immobilized antibodies, and a secondary antibody labeled with horseradish peroxidase, enabled colorimetric detection. Optical density was measured at a wavelength of 450 nm after the addition of the stop solution.

### 4.8. The Caspase-Glo^®^ 8 and 9 Assay

The detection of Caspase 8 and 9 activations in MCF-7 cells was carried out using the Caspase-Glo 8 and 9 luminometric assay kit (Promega, Mannheim, Germany), following the manufacturer’s protocol. The assay kit provides luminescent Caspase 8 and 9 reagents, emitting signals upon cleavage. Initially, MCF-7 cells were seeded in 96-well plates at a density of 2 × 10^4^/mL in 100 μL of standard culture medium and allowed to grow for 24 h. Subsequently, the cells were treated with U-359, Ox, 5-Fu alone, or with U-359 in combination with Ox or 5-FU at their IC_50_ concentrations for 24 h. Following this incubation, the 50 μL of Caspase-Glo^®^ Reagents were added. Finally, the plate was gently mixed for 2 min, incubated for 30 min at room temperature, and then read within 2 min using Flexstation 3. A comparison of combination treatment effects was performed against individual compound treatments.

### 4.9. Quantitative Real-Time PCR Assay

The quantitative RT-PCR was employed to analyze mRNA levels of Bax, Bcl-2, p-53, p21, and Caspase 3 genes. MCF-7 cells were seeded at a density of 4.0 × 10^5^ cells/well in 6-well plates and treated with U-359, Ox, 5-Fu alone, and with U-359 in combination with Ox or 5-FU at their IC_50_ concentrations for 24 h. The effects of the combination treatment were compared with individual compound effects. Total RNA extraction was performed using the Total RNA Mini Kit (A&A Biotechnology, Gdynia, Poland). RNA concentration was determined using a sensitive single-tube fluorimeter. Subsequently, cDNA synthesis was carried out using the Transcriba Kit (A&A Biotechnology, Gdynia, Poland). The amplification of gene-specific primers was conducted using Real-Time 2x-PCR SYBR Master Mix (A&A Biotechnology, Gdynia, Poland) in a Stratagene MX3005P QPCR System (Agilent Technologies, Inc., Santa Clara, CA, USA) following the manufacturer’s instructions. The normalization of gene expression employed GAPDH as an internal reference gene, and gene expression levels were calculated using the 2^−∆∆CT^ method [23].

### 4.10. Assessment of ABCB1, ABCG2, and NF-κB/p65 Protein Levels by ELISA-Based Method

To quantify ABCB1, ABCG2, and NF-κB/p65 protein levels in MCF-7, ELISA-based assays were conducted using specific kits from Finetest BT LAB (Shanghai, China). Cells were plated in 6-well plates at a density of 5.0 × 10^5^ cells per well and allowed to adhere for 24 h. Afterward, the cells were treated with U-359, Ox, 5-FU, and with U-359 in combination with Ox or 5-FU, at their respective IC_50_ concentrations for an additional 24 h. Following treatment, cells were washed with PBS, centrifuged, and lysed according to the manufacturer’s instructions. Appropriately diluted protein extracts (50 μg) and standards were added to 96-well plates coated with specific antibodies for ABCB1, ABCG2, and NF-κB/p65. The captured proteins were then detected using a secondary antibody conjugated with horseradish peroxidase (HRP), leading to a colorimetric signal. The optical density (OD) of the resulting yellow solution was measured at 450 nm to quantify protein levels.

### 4.11. The Bliss Independence Model

Synergy indices (SI) were computed to evaluate the synergistic, additive, or antagonistic effects of Ox or 5-FU in combination with the modulator U-359 on MCF-7 cells using the Bliss Independence Dose–Response Surface Model.

Such a model is a mathematical framework used to analyze drug interactions, particularly in the context of combination therapies. This model assesses whether the combined effect of two drugs is greater (synergistic), equal to (additive), or less (antagonistic) than the expected effect based on the individual responses of each drug.

For each combination of Drug A and Drug B, the Bliss Independence Model was applied to predict the expected response based on the individual drug responses: EAB = EA + EB − EA × EB, where EAB is the expected effect of the combination, EA is the effect of Drug A alone, and EB is the effect of Drug B alone. The observed effect of the drug combination was compared to the predicted effect using the Bliss Independence Model. Synergy is indicated by an SI value less than 1, additivity by an SI value equal to 1, and antagonism by an SI value greater than 1 [24].

### 4.12. Statistical Analysis

Statistical evaluation was carried out with Prism 6.0 (GraphPad Software Inc., San Diego, CA, USA). Data, representing a minimum of three independent experiments conducted in triplicate, were presented as mean ± SEM. Significance was determined using a one-way ANOVA followed by post hoc multiple comparisons utilizing the Student–Newman–Keuls test. Statistical significance levels were denoted as * *p* < 0.05, ** *p* < 0.01, and *** *p* < 0.001.

## 5. Conclusions

This study provides compelling evidence supporting the efficacy of U-359 in combination with Ox or 5-FU, underscoring its potential as a modulator in breast cancer therapy. By enhancing apoptotic signaling and modulating drug resistance mechanisms, U-359 presents a promising approach for overcoming chemotherapy resistance. Furthermore, the mechanistic insights gained, including the regulation of apoptotic pathways and MDR markers, contribute to a deeper understanding of U-359’s mode of action. Future investigations will focus on evaluating its efficacy in MCF-7 cell lines resistant to Ox and 5-FU, facilitating the optimization of U-359-based therapeutic strategies and assessing its applicability across various cancer types.

## Figures and Tables

**Table 1:** mRNA expression levels of apoptosis-related genes in MCF-7 cells treated with U-359, Ox, 5-FU, U-359+Ox, or U-359+5-FU for 24 h.

	Gene				
Compounds	*Bax*	*Bcl-2*	*p21*	*p53*	*Caspase3*
U-359	2.9 ± 0.3 ***	0.003 ± 0.00004 ***	60.5 ± 0.3 ***	1.2 ± 0.08 *	10.0 ± 0.4 ***
Ox	0.14 ± 0.008 ***	2.4 ± 0.2 *	0.021 ± 0.002 ***	0.56 ± 0.004 ***	1.3 ± 0.04 *
5-FU	0.17 ± 0.01 ***	1.5 ± 0.004 ***	0.31 ± 0.02 ***	0.5 ± 0.001 ***	0.3 ± 0.04 ***
Ox+U-359	2.5 ± 0.09 ***	0.02 ± 0.0001 ***	2.4 ± 0.2 ***	19.3 ± 0.7 ***	8.1 ± 0.4 ***
5-FU+U-359	1.9 ± 0.2 ***	0.14 ± 0.008 ***	16 ± 0.1 ***	13.9 ± 0.5 ***	5.04 ± 0.3 ***

Data represent mean ± SEM of three independent experiments performed in triplicate. *** *p* < 0.001, * *p* < 0.05.

**Table 2:** mRNA expression levels of resistance-related genes in MCF-7 cells treated with U-359, Ox, 5-FU, U-359+Ox, and U-359+5-FU for 24 h.

	Gene		
Compounds	*ABCB1*	*ABCG2*	*NF-kb*
U-359	0.01 ± 0.001 ***	0.01 ± 0.002 ***	0.8 ± 0.03 ***
Ox	38.0 ± 4.8 ***	5.3 ± 0.3 ***	21.3 ± 0.2 ***
5-FU	1.28 ± 0.004 *	1.45 ± 0.2 ***	1.9 ± 0.02 ***
Ox+U-359	0.007 ± 0.0 ***	0.16 ± 0.001 ***	0.003 ± 0.0 ***
5-FU+U-359	0.25 ± 0.01 ***	0.03 ± 0.004 ***	0.39 ± 0.04 ***

Data represent mean ± SEM of three independent experiments performed in triplicate. *** *p* < 0.001, * *p* < 0.05.

**Table 3:** Synergistic effects analysis using the Bliss Independence Dose–Response Surface Model.

Protein	Combination	Synergy Index (SI)	Interpretation
ABCB1	Ox+U-359	-	U-359 did not influence the level of ABCB1 protein
	5-FU+U-359	-	U-359 did not influence the level of ABCB1 protein
ABCG2	Ox+U-359	0.29 ***	U-359 partially reversed Ox-induced up-regulation of ABCG2, indicating moderate synergy (SI < 1)
	5-FU+U-359	0.07 ***	U-359 significantly reduced ABCG2 expression in the presence of 5-FU, demonstrating moderate synergy (SI < 1)
NF-κB	Ox+U-359	0.69 ***	U-359 significantly reduced NF-κB expression in the presence of Ox, demonstrating moderate synergy (SI < 1)
	5-FU+U-359	0.17 ***	U-359 enhanced the efficacy of 5-FU by reducing NF-κB level, reflecting moderate synergy (SI < 1)

Data represent mean ± SEM of three independent experiments performed in triplicate. *** *p* < 0.001.

## Data Availability

The original contributions presented in this study are included in the article. Further inquiries can be directed to the corresponding author.

## Abbreviations

ABC	ATP-binding cassette
ABCB1	ATP-binding cassette subfamily B member 1 (P-glycoprotein)
ABCG2	ATP-binding cassette subfamily G member 2 (breast cancer resistance protein, BCRP)
5-FU	5-Fluorouracil
MDR	Multidrug resistance
NF-κB	Nuclear factor kappa B
Tx	Taxol
U-359	3-p-bromophenyl-1-ethyl-5-methylidenedihydrouracil
MCF-7	Michigan Cancer Foundation-7 (breast cancer cell line)
Ox	Oxaliplatin
